# TakoTsubo Syndrome: A Well-Known Disease but Not Everything Is Clear Yet

**DOI:** 10.31083/j.rcm2306184

**Published:** 2022-05-25

**Authors:** Cesare de Gregorio, Lorenzo Pistelli, Marco Borgi, Olimpia Trio, Yoshihiro J Akashi, Giuseppe Andò

**Affiliations:** ^1^Department of Clinical and Experimental Medicine, Cardiology Section, Azienda Ospedaliera Universitaria Policlinico “Gaetano Martino”, University of Messina, 98124 Messina, Italy; ^2^Division of Cardiology, Department of Internal Medicine, St. Marianna University School of Medicine, 216-8511 Kawasaki, Japan

**Keywords:** TakoTsubo Syndrome, acute coronary syndrome, stress cardiomyopathy, catecholamine, heart failure, left ventricular dysfunction

## Abstract

TakoTsubo Syndrome (TTS) is a stress-induced cardiac disease characterized by temporary and segmental left ventricle dysfunction, typically involving the apex. Post-menopause women are more frequently affected. ECG and clinical features at presentation may be similar to those observed in acute coronary syndrome (ACS). However underlying pathomechanisms are completely different and, for what concerns TTS, extremely debated and not yet completely understood. Some hypotheses have been proposed during years, mostly regarding catecholamine-induced cardiotoxicity and microvascular dysfunction, usually following a trigger event which may be either “emotional” (primary TTS) or “physical” (secondary TTS). Additional modulators like neuroendocrine disorders (particularly hypothalamic-pituitary-adrenal axis dysfunction and estrogen drop in menopause) may play a crucial role in TTS onset. Despite being originally considered more benign than ACS, several studies have enlightened that TTS and STEMI are burdened by the same in-hospital mortality and complications. However, TTS and ACS complications somehow differ for what concerns incidence, the underlying mechanisms, and both long- and short-term outcomes. Full recovery in TTS requires weeks to months and cases of recurrences have been described, but no single clinical feature seems to predict subsequent episodes so far. By now, apart from inhibitors of the Renin-Angiotensin-Aldosterone System (RAASi), no drug has proved to be effective either in the acute or chronic phase in reducing mortality, improving outcome, or preventing recurrences.

## 1. Introduction 

TakoTsubo cardiomyopathy, more recently preferred as a *syndrome* (TakoTsubo Syndrome, TTS), is a cardiac disease characterized by temporary hypokinesis, dyskinesis, or akinesis in the left ventricle (LV) wall segments with (more frequent) or without apical involvement, exceeding a single coronary vessel blood flow distribution. Women are more susceptible to TTS, and a stress elicitation (personal or social occasion, as well as acute disease) is the most common trigger [1, 2, 3, 4, 5].

Angiography usually shows no significant coronary artery disease (CAD), but a coexistence of bystander CAD and TTS is possible. Relevant abnormalities are commonly observed on the electrocardiogram (ECG) such as ST-segment elevation and/or T-wave inversion, as well as prolonged QT interval, mimicking a typical acute coronary syndrome (ACS) [1, 3, 4, 6].

## 2. Pathophysiology

The precise pathomechanism of TTS is still uncertain and probably multifactorial. Many hypotheses have been linked to the occurrence of cardiomyopathy. Both physical and emotional stress can trigger the onset of the syndrome. Either physical or emotional stress has been reported in 60–80% of patients. There is another 20–30% in whom no triggers were identified. The most common physical stressors included surgery, infections, and acute respiratory failure. Emotional triggers were the death of a loved one, relationship conflicts, fear, anger, and anxiety depicts the recent inter-TAK classification [1, 2, 3, 7].

The most accepted pathogenetic theories are catecholamine-induced cardiotoxicity and microvascular dysfunction, but additional modulators like neuroendocrine disorders, dysfunctional cognitive and emotional brain centers, especially of the hypothalamic-pituitary-adrenal axis, have been outlined [1, 3, 5, 8, 9, 10].

The plasma levels of epinephrine were critically elevated in many patients with emotional stress in a study by Wittstein [11]. Authors also found that serum catecholamine concentration was 2- to 3-fold higher in this clinical setting than in typical ACS patients.

A catecholaminergic mechanism was hypothesized in past studies and confirmed by reviews and consensus statements [1, 2, 4, 5, 12]. This theory subtends similar features observed in exogenous catecholamine administration and pheochromocytoma, which, however, TTS has been established to be discerned from, though there is a lack of agreement on this aspect in the scientific community [9, 10, 11, 13, 14, 15, 16, 17, 18].

The abnormal distribution of catecholamine receptors in the myocardium was highlighted by Lyon *et al*. [19] in 2008. The elevated concentration of norepinephrine, released by the sympathetic system, showed high affinity to β2 receptors (β2ARs, with negative inotropic and lusitropic response) than β1 receptors (β1ARs, with positive response) in TTS patients. Being myocardial concentration of β2ARs in humans higher than in other mammals, it was supposed that overexpression of β2ARs in the apical wall of predisposed individuals may be interpreted as a protective response against the catecholaminergic (epinephrine-related) myocardial distress [1].

More recently, G protein-coupled kinase 2 and β-arrestin 2 were demonstrated to initiate the alteration of βARs signaling in TTS patients. Both receptors were overexpressed on the cardiomyocyte membrane as by immunohistochemistry analysis, especially in the acute phase of LV dysfunction. TTS subjects showed much higher signaling than patients with dilated cardiomyopathy and controls, and sequential biopsies revealed that membrane expression of such receptors faded over time [20].

About 90% of patients with TTS are (postmenopausal) women, but the syndrome also affects men, especially in Japan [21]. For that reason, the pathogenetic role of estrogens has also been widely studied in this clinical setting [22, 23, 24].

The risk of experiencing TTS was higher in patients carrying anomalous T-allele of the gene encoding estrogen receptor 1, but confirmatory studies are still lacking [25]. In fact, though prior evidence, hormone replacement therapy was not protective against the development of TTS, leading researchers to conclude that estrogenic deficiency or anomalous receptors are possible drives of the syndrome [21, 22, 23, 25].

Small coronary artery and microvascular dysfunction might also play a role in precipitating the syndrome. Studies have revealed the incidence of TTS in women resembling that of migraine headache and the Raynaud phenomenon [26]. Then, vascular dysfunction may be a common feature in such patients. Accordingly, a catecholamine-based microvascular spasm was also thought to cause a transient drop in coronary blood flow reserve and ensuing apical ballooning phenomenon [27].

The same functional impairment may occur during histaminergic distress caused by an anaphylactic response to drugs, foods, or contrast agents [28]. In some patients, a combination of TTS and type-1 Kounis syndrome was hypothesized [29, 30, 31], as demonstrated by Desay *et al*. [32] from the US National Inpatient Sample in a 7-year period (2007–2014). These authors identified such a combination in African and Asian elderly females the most, also suffering from hypertension in 100% and dyslipidemia in 62% of cases.

Interestingly, studies have demonstrated a seasonal variability in the occurrence of TTS. More cases were observed in summer (30%) than in winter (18%) in 260 consecutive patients (95% women) from a New Zealand study, compared to the highest occurrence of ACS in winter [33]. A recent multicenter study in Japan also confirmed such a trend that demonstrated seasonal variation only in the female group [34]. The authors reported more events from July to December, especially in the afternoon. These results also suggest that the pathogenesis and clinical features of TTS might therefore differ according to climate.

## 3. In-Hospital Outcomes

Despite being originally considered less malign than ACS, several studies have enlightened that TTS is burdened by the same in-hospital mortality rate as in ST-elevation myocardial infarction (STEMI), with overall occurrence as by 4–5% of patients admitted for chest pain [1, 4, 35]. It is not surprising that these two conditions share some predisposing factors (smoking habit and endothelial dysfunction appeared to be important aspects in both diseases) and complications [36]. However, patients with ACS and TTS are substantially different for what concerns sex, age, CV risk factors, and comorbidities, as the latter are more likely to be women, have younger age, and have fewer comorbidities). It is interesting to note that, despite indicators of poor outcome (male sex, age >70 years, and physical illness/comorbidity) are more consistent in ACS patients, both conditions are burdened by the same in-hospital complications, and similar short- and long-term outcomes [37, 38, 39]. In this regard, it has recently been shown that the GRACE score can be used in patients with TTS to predict the risk of short- and long-term mortality [40].

Furthermore, it is crucial to differentiate primary from secondary TTS. Most primary TTS affect postmenopausal women without coronary artery disease (CAD); in this case, moderate LV dysfunction is probably caused by microvascular dysfunction during strong emotional stress (Table 1, Ref. [41]). In secondary TTS, on the other hand, severe LV dysfunction is triggered by physical stress. Previous studies reported that most in-hospital TTS are secondary TTS, which are strongly comorbidities-related and burdened by higher in-hospital mortality compared with out-of-hospital TTS (more likely to be primary TTS) [42]. There is a discordance between European and Japanese registries for what concerns in-hospital outcome: in-hospital mortality appears to be higher among Japanese patients, however, ethnicity itself is not likely to play a role in increasing mortality [43].

**Table 1.** **Differences between Primary and Secondary TTS**.

	Primary	Secondary
Gender	Women (>50 years)	Women/Men
Stressor(s)	Emotional	Physical/Organic
CAD	Absent	Possible
Underlying pathomechanism	Microvascular/allergic dysfunction	Micro/macrovascular dysfunction
LV dysfunction	Moderate-to-severe	Often severe
LV complications	Uncommon	Frequent
LV functional recovery	Short term	Mid-late term
Prognosis	Variable (usually benign)	Variable (often poor)
Recurrences	Likely	More likely

Modified from Galiuto *et al*. [41]. CAD, Coronary Artery Disease; LV, Left Ventricle.

### 3.1 Differences between ACS and TTS

**Fig. 1.**
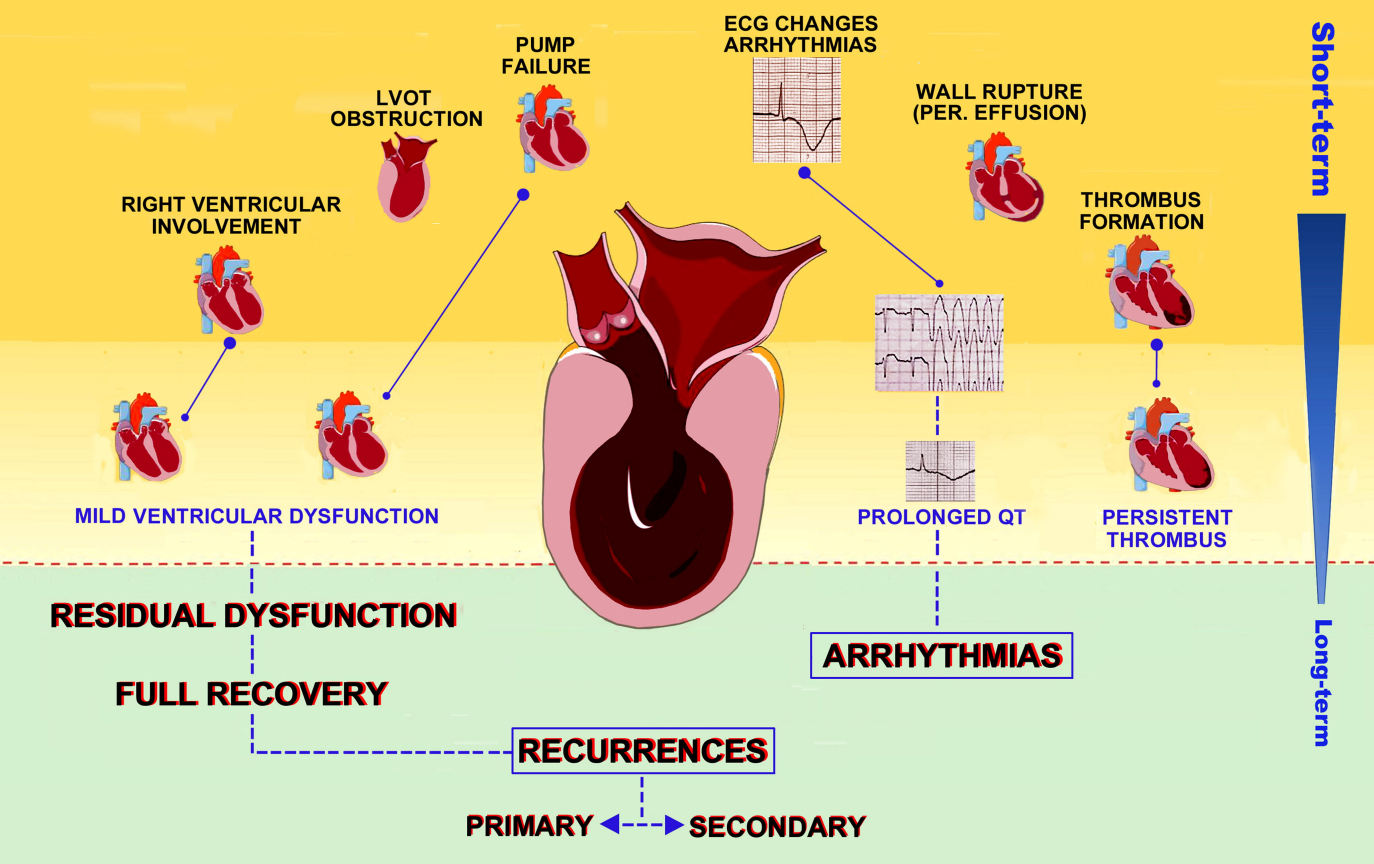
**Clinical outcomes in TTS patients**. Possible complications of TTS are depicted according to timing of occurrence, from the inpatient phase to long term outcomes and recurrences.

During hospitalization, 32.9% of TTS patients are estimated to have complications [44]. The most frequent adverse outcome in both conditions is left ventricular dysfunction, affecting approximately 20% (12–45%) of TTS patients and 13–32% of STEMI patients [45, 46, 47]. However, in acute myocardial infarction (AMI) LV failure is usually due to inotropism loss or acute mitral regurgitation, while in TTS (Fig. 1) up to 25% of heart failure (HF) are a consequence of left ventricular outflow tract obstruction (LVOTO) determined by hyperdynamic proximal LV chamber and systolic anterior motion (SAM) of the anterior mitral valve leaflet [36].

Cardiogenic shock precipitates the acute disease in approximately 20% of TTS and 6–10% of STEMI [4, 48]. There is no unanimous consensus about the best strategy to treat cardiogenic shock in TTS, but previous studies showed that TTS patients are more likely to require mechanical respiratory support than ACS [49]. Notwithstanding, the mortality rate is substantially higher among cardiogenic shock due to STEMI (>50%) vs TTS (15%) [39, 50]. As previously stated, this difference may be due to the patient underlying condition at admission.

Left ventricular ejection fraction (LVEF) on admission is an important predictor of mortality in both TTS and ACS. In comparison to average ACS, TTS patients usually show significantly lower EF, but no differences were enlightened when compared with STEMI [45, 46, 47, 48, 50].

Supraventricular arrhythmias, in particular atrial fibrillation, are associated with a poor prognosis and a higher number of compliances in both TTS and STEMI [45, 51]. Atrial fibrillation onset is probably the result of high catecholamine release in addition to myocardium inflammatory state and atrial overload resulting from acute mitral insufficiency and LV dysfunction [45, 51].

Originally, ***ventricular arrhythmias*** have been described to occur with the same incidence (8–9%) in patients with TTS and AMI [35, 47, 52]. However, it is now believed that ventricular arrhythmias are less frequent in TTS than in AMI. Syed *et al*. [52] reported a prevalence of 3.4% of ventricular tachycardia or fibrillation, while it is proposed that 6–8% of patients with AMI develop malignant ventricular arrhythmias during the acute phase. This may be due to β2ARs switching from Gs to Gi in TTS. This change, which has been described as a co-participant factor in myocardial catecholamine-induced stunning, may play a protective role in catecholamine-triggered arrhythmias. Moreover, acute ischemia determines cells’ ***electrical instability***, leading to abnormal myocardial depolarization and arrhythmias. The most largely described ECG pattern in TTS patients is QT interval prolongation. However, this feature can also be found in STEMI patients, due to abnormal autonomic modulation and epicardial to endocardial electrical gradient. QTc >500 ms with giant negative T waves and J point elevation is described as the main predictors for TdP, requiring external defibrillation in 4.9% [51, 53].

Mechanical complications, such as septal perforation, occur rarely, but consequences are extremely serious. Scant data is available due to the rarity of this complication, however, the mortality rate in TTS was assessed around 83%, reaching 90% during the first 8 days [54]. It has been suggested that high intraventricular pressure and high LV wall stress may be the major determinants [54]. It is interesting to observe how, despite the different etiology, in MI mechanical complications occur within the same time window (from 24 hours to a few weeks for septum and from 2 to 7 days for papillary muscles) and are burdened by comparable mortality (20–75%) [45]. Apical akinesia is a frequent issue in both conditions and is the main determinant of LV thrombosis. Intraventricular thrombosis occurs in approximately 5% of either TTS and STEMI patients of both TTS and ACS, leading to embolic stroke in 1–5% cases among TTS patients [4, 45, 47].

### 3.2 In-Hospital Stay 

A Portuguese study assessed in-hospital stay lasting 5 ± 6 days [44]. However, defining precise duration is difficult. Hospitalization time is extremely variable, depending on complications that occurred and patient performance status at presentation. As previously stated, TTS in hospitalized patients is burdened by a higher complication rate and consequently, the in-hospital stay is longer for these patients. As well, being associated with greater hemodynamic instability, right ventricle (RV) involvement determines a longer hospital stay [12, 55]. Thus, the discharge time for TTS patients is challenging and dependent on its complications. Hemodynamic status and arrhythmic profile, along with markers monitoring (especially BNP levels) are the main determinants of a safe discharge [55].

### 3.3 Thrombus Formation and Cardioembolic Events

Ventricular thrombus is a fearsome complication that might occur in all those clinical conditions causing ventricular dysfunction, such as dilated cardiomyopathy and AMI. TTS is of no exception in this respect, as it shows an increased likelihood of forming an LV thrombus with an estimated incidence of around 2–8% [56, 57, 58].

Patients who suffered from thromboembolic complications showed a significantly higher mortality rate (*p* = 0.02) over a mean follow-up of 3 years in a recent prospective study [59]. Thrombi generally occur 2–5 days after TTS onset and appear to be associated with both elevated C-reactive Protein and ST-segment elevation, despite the latter being present only in nearly half of patients with LV thrombosis [59]. Regional hypokinesia, endothelial activation, systemic hypercoagulability, and blood stasis due to myocardial stunning all combine to constitute a pro-thrombotic state, especially in patients presenting with apical type TTS, advanced age, and late hospital presentation [60, 61].

### 3.4 Left Ventricular Functional Recovery 

Cardiac alterations in TTS are both regional wall-motion and electrocardiographic abnormalities. Despite the former normalizes within a few weeks, the latter persist for several months [55]. As long as myocardial edema persists, T-wave inversion is evident, making such T-waves alteration an indirect index of myocardial edema [58]. However, ventricular arrhythmias usually occur only in the acute phase and so an implantable cardioverter-defibrillator is not indicated despite ECG abnormalities persisting in the subacute phase [55].

Usually, in TTS LV motion abnormalities recover up to normality (in both systolic and diastolic function) in 4 to 8 weeks [55]. Early signs of LV functional improvement can be recognized by analyzing the changes in global longitudinal strain and apical twisting/untwisting [55, 62, 63]. Improvement in global longitudinal strain (GLS) is associated with a reduction (up to a complete resolution) of LVOTO and mitral insufficiency [62]. It is curious to observe how improvement is not simultaneous for all myocardial segments, showing a different susceptibility to catecholamine insult and stunning [62]. This aspect should be further investigated, as it may reveal important implications to better understand the pathophysiology of disease [36, 55].

### 3.5 Right Ventricular Functional Recovery 

Up to 35% of TTS patients present with right ventricular dysfunction, often clinically silent [10]; however, biventricular involvement predicts a worse outcome [12, 61]. Biventricular involvement is typically identified on imaging studies such as echocardiography and MRI. In observational retrospective studies, the most frequently affected segments of the right ventricle were the apical-lateral, anterolateral, and inferior walls [21]. This involvement has been associated with a higher prevalence of in-hospital major adverse cardiovascular events, including heart failure, bilateral pleural effusion, cardiogenic shock, and in-hospital mortality, especially in older patients with low LV ejection fraction [21, 32].

## 4. Long-Term Outcomes

Although TTS has long been considered a benign disease, recent observational registries seem not to confirm such a trend [62]. In a large retrospective study in 1750 patients, long term follow-up showed a patient-year risk of all-cause death of 5.6%, with a 9.9% per patient-year risk of major adverse cardiac, including death from any cause, recurrence of TTS, stroke or TIA, or AMI [21]. When compared to age- and sex-matched STEMI/NSTEMI patients, TTS showed similar long-term outcomes [62]. Among clinical determinants, male sex, diabetes mellitus, and Killip class III/IV at presentation were proven to significantly affect long-term prognosis [64]. Although sex-related differences in prognosis (poorer in men) have also been demonstrated,, information about the exact prognosis is still lacking, also because of ethnic disparities in both clinical characteristics and in-hospital outcomes [43].

### 4.1 Ventricular Dysfunction on Echocardiography

The central role of cardiovascular imaging in the diagnosis of TTS is well established. A growing body of evidence demonstrated that transthoracic echocardiography, as well as being of great help to the clinician in the differential diagnosis of TTS, provides independent and incremental long-term prognostic value in addition to other clinical factors. Severely depressed LVEF (<35%) in patients enrolled in the Takotsubo Italian Network (TIN) on hospital admission resulted in poor outcomes, as well as a higher risk of MACE (HR 2.184, 95% CI 1.231–3.872) during long term follow-up [65]. Right ventricular dysfunction as well is not uncommon and should always be characterized by imaging studies as it has been shown to significantly worsen long-term prognosis (HR 2.73, 95% CI 1.13–6.62, *p* = 0.026) [66].

As in other cardiovascular diseases, conventional transthoracic echocardiography may benefit from the use of advanced techniques such as speckle tracking to better estimate ventricular function, providing incremental prognostic value. In a recent study involving 650 TTS patients, Akashi and colleagues acquired LV GLS at presentation in addition to LVEF [67]. At follow-up, long-term mortality significantly differed among different quartiles depending both on the baseline LVEF (*p *< 0.001) and LV GLS (*p *< 0.001). Such a result is likely to show how useful both measurements can prove in refining long-term risk when added to well-established clinical determinants of prognosis such as age, male sex, ST-T changes, and type of triggers [67].

A recent prospective study by Eitel and colleagues demonstrated that LV function, even if markedly reduced at presentation, fully recovered after days or months from the index event, as by echocardiography and/or MRI with a median time of 97 days (IQR, 36–123) [68].

### 4.2 Arrhythmic Events

TTS has long been considered a pro-arrhythmic condition, and cardiac arrhythmias are certainly among the most feared long-term complications, due to their severity and unpredictability, with an incidence that has been estimated from 7 to 14% [59, 69]. Among a cohort of 906 patients, El-Battrawy *et al*. [70] recognized significantly higher 30-day mortality rates in those who had arrhythmic disorders compared to non-arrhythmic patients (log-rank <0.01). Each arrhythmia affects prognosis differently, as sustained ventricular tachycardia (VT) proved to be related to a worse prognosis when compared to nonsustained VT, as well as monomorphic to polymorphic VT [69].

Concerning mechanisms, corrected QT (QTc) prolongation has been reported in different cohorts of patients, affecting up to one-half of the patients at presentation, and a QTc >500 ms has been found in most of the patients experiencing a malignant ventricular arrhythmia, behaving like an acquired long QT syndrome [21].

While myocardial fibrosis is a common finding in ischemic heart disease (IHD), with re-entrant arrhythmias stemming mostly from altered myocardial conduction patterns around ischemic scars and fibrotic areas, TTS does not seem to share such a pathological substrate. Indeed, in a large study involving 256 patients with TTS, Eitel and colleagues demonstrated minute focal or patchy nonischemic myocardial scarring at cardiac MRI in 9% of such cases, but using a much lower threshold of signal intensity and a smaller extent of late gadolinium enhancement when compared to IHD [64].

Differently from IHD, investigators found myocardial edema to be widely present in TTS patients on admission, with a prevalence of 162 out of 199 patients (81%), and a regional distribution consistent with the pattern of LV dysfunction, suggesting a role in the delayed and dispersed ventricular repolarization reflected on ECG by a prolonged QTc interval [68].

### 4.3 Treatment of Thrombus Formation

Anticoagulation in case of thrombus formation is the only therapy that proved to be effective and should be promptly started in the absence of high bleeding risk, and generally discontinued after 3 months or after echocardiographic demonstration of LV thrombus resolution and LVEF recovery at follow-up [5]. Despite the lack of evidence, prophylactic anticoagulation should be considered in patients with a severely reduced LVEF or apical akinesia on admission to hospital, with an individualized approach, as not all patients would benefit the same, depending on the risk-benefit profile [62].

However, long-term prognostic studies describing outcomes in TTS patients complicated by a ventricular thrombus, as well as those with cardioembolic events on admission, are lacking yet. In the acute phase, prothrombotic state was described as the result of platelet activation, peripheral vasoconstriction, and, of course, ventricular dysfunction, justifying a possible role of antiplatelet therapy in preventing major events in these patients.

Treatment with aspirin did not prove to reduce the risk of major adverse cardiovascular events in TTS patients in both short- and long-term follow-up [71].

## 5. Recurrences

Recurrent TTS after completely recovered LV dysfunction is still under investigation [72]. Current literature suggests a recurrence rate of 8%, with a variability ranging from 1 to >20%, depending on the study population and follow-up [1, 12, 47, 73].

Recurrences were seen within the first 4 years in patients younger than 50 years at the first event. Their mortality rate was up to 2.7% at 5 years, strictly dependent on comorbidities and sex [12, 72, 73]. In a recent population-based cohort study on 519 US patients, recurrence of syndrome occurred in 7.5% of patients over a median of 5.2 years of follow-up. Authors found a higher proportion of elderly men with a 2.5-fold higher risk of death or recurrence [74].

Overall, no sure clinical features were recognized to predict subsequent episodes [72]. Like in AMI patients, the persistence of predisposing factors (diabetes and hypertension first) is the major determinant, but comorbidities are also reported to be potential triggers as well (Table 2, Ref. [73, 74, 75, 76]). Conversely, the impact of therapy is controversial and recurrence among TTS is substantially lower when compared to AMI, more likely to occur in primary TTS, which are subjected to psychological triggers. However, intercurrent diseases may be potential triggers, and this may suggest an overlapping between primary and secondary forms [62, 72, 73, 77].

**Table 2.** **Clinical characteristics of patients with recurrence of TTS according to different studies [73, 74, 75, 76]**.

Clinical characteristic	
Female gender	>85%
Timing range (days)	30–3600
Classical risk factors	Diabetes, Hypertension
P/N disorders	35–45%
NC triggering diseases	Endocrine, Infective, Neurologic, Renal, Respiratory, Others
LVD pattern (vs index event)	Similar (60–70%), Different (30–40%)
Multiple recurrences	Rare
BB therapy (prior recurrence)	60–80%

BB, Beta-Blocker; LVD, Left ventricle dysfunction; NC, Non-cardiac; P/N, Psychiatric/neurological.

Cases of multiple recurrences in the same patient have also been described, but the triggering event and the ballooning pattern may be different in each recurrence [36, 55, 62, 72]. Therefore, better identification of the stressors may help prevent further events, especially for what concerns emotional triggers [65, 72]. A targeted therapy, not only pharmacological but also stress-containing, anti-depressant, and for migraine, may help prevent future episodes [65].

Also, there is lacking unanimity on whether cardio-active therapy (particularly beta-blockers) may be useful to contrast the effects of catecholamine excess and then prevent subsequent events. Although beta-blockers appear to be the most intuitive prevention choice for recurrences, approximately 70% of recurrent-TTS patients were already on this therapy [55]. In the study of Lau *et al*. [74], beta-blocker exposure was associated with lower mortality and recurrences, while there was no association with ACE-inhibitors or ARBs. Conversely, ACE-inhibitors and ARBs have been demonstrated to be more effective against catecholaminergic damage, inflammation, and fibrosis [4, 5].

We are aware of the efforts to draw definite conclusions to avoid recurrences, but further evidence is needed first because most observational studies have been differently designed and managed, and important differences could also be related to the racial make-up of the study population.

On the basis that catecholaminergic tone may affect platelet activation, some authors advanced the hypothesis of a preventing role for Aspirin also in TTS patients, but recent studies did not prove its effectiveness in short- or long-term outcomes [5, 57, 71].

## 6. Conclusions

In recent years, important clinical registries and international trials enriched our knowledge on both the acute phase and long-term mortality related to TTS, raising awareness of this multifaceted and complex clinical disorder. Pathomechanism, clinical and prognostic features of TTS were summarized in the light of current literature.

Primary TTS seems to have a better prognosis, whereas secondary forms get worse outcomes, outpacing ACS. Recurrences are rare but still unpredictable, and blockade of the Renin-Angiotensin-Aldosterone system remains the primary therapeutic target for prevention.

Further research is encouraged to shed further light on the complex pathophysiology of TTS, define sure prognosticators and more effective treatments against recurrences.

## Abbreviations

ACE, Angiotensin-Converting Enzyme; ACS, Acute Coronary Syndrome; AMI, Acute Myocardial Infarction; ARB, Angiotensin Receptor Blockers; BNP, Brain Natriuretic Peptide; CAD, Coronary Artery Disease; ECG, electrocardiogram; EF, Ejection fraction; GLS, Global Longitudinal Strain; HF, Heart Failure; IHD, Ischemic Heart Disease; LV, Left Ventricle; LVEF, Left Ventricular Ejection Fraction; LVOTO, Left Ventricular Outflow Tract Obstruction; MACE, Major Adverse Cardiovascular Events; MI, Myocardial Infarction; MR, Mitral Regurgitation; MRI, Magnetic Resonance Imaging; NSTEMI, Non-ST-Elevation Myocardial Infarction; QTc, Corrected QT; RV, Right Ventricle; SAM, Systolic Anterior Motion; STEMI, ST-Elevation Myocardial Infarction; TIA, Transient Ischemic Attack; TIN, Takotsubo Italian Network; TT, TakoTsubo; TTS, TakoTsubo Syndrome; VT, Ventricular Tachycardia.
